# Interactions between rat alveolar epithelial cells and bone marrow-derived mesenchymal stem cells: an in vitro co-culture model

**DOI:** 10.1186/s40635-015-0053-2

**Published:** 2015-05-24

**Authors:** Hiroyuki Ito, Tokujiro Uchida, Koshi Makita

**Affiliations:** Department of Anesthesiology, Graduate School of Medical and Dental Sciences, Tokyo Medical and Dental University, 1-5-45 Yushima, Bunkyo-ku, Tokyo 113-8519 Japan

**Keywords:** Bone marrow-derived mesenchymal stem cell, Alveolar epithelial cell, Three-dimensional structures, Co-culture, Receptor for advanced glycation end-products, Surfactant protein D

## Abstract

**Background:**

Bone marrow-derived mesenchymal stem cells (BMSCs) reduced the severity of acute lung injury after transplantation in multiple experimental studies, and several paracrine soluble factors secreted by the cells likely contribute to their therapeutic effect. The direct interactions between BMSCs and alveolar epithelial cells (AECs) may be an important part of their beneficial effects. Therefore, we assessed the interactions between BMSCs and AECs using a co-culture model of these two cell types from rats.

**Methods:**

BMSCs and AECs were co-cultured using a Transwell system under the following conditions: (1) separated co-culture—AECs seeded on the insert and BMSCs in the base of the well; and (2) mixed co-culture—AECs on top of the monolayer of BMSCs on the culture insert and no cells in the base of the well. After 21 days of culture, the cells on the membrane of the culture insert were fixed and stained with antibodies against the receptor for advanced glycation end-products (RAGE), surfactant protein D (SP-D), and zona occludens protein-1, and then analyzed by confocal microscopy.

**Results:**

In the separated co-culture condition, the phenotype of the AECs was maintained for 21 days, and cluster formation of SP-D-positive cells was induced in the AEC monolayer. We also found cluster formations of phospholipid-positive cells covered with RAGE-positive epithelial cells. In the mixed co-culture condition, the BMSCs induced alveolar-like structures covered with an epithelial cell layer. To determine the effect of keratinocyte growth factor (KGF) on this three-dimensional structure formation, we treated the mixed co-cultures with siRNA for KGF. While KGF siRNA treatment induced a significant reduction in surfactant protein transcript expression, formation of the alveolar-like structure was unaffected. We also assessed whether Gap26, a functional inhibitor of connexin-43, could mitigate the effect of the BMSCs on the AECs. However, even at 300 μM, Gap26 did not inhibit formation of the alveolar-like structure.

**Conclusions:**

BMSCs release soluble factors that help maintain and sustain the AEC phenotype for 21 days, and direct interaction between these two cell types can induce a cyst-like, three-dimensional structure covered with AECs.

**Electronic supplementary material:**

The online version of this article (doi:10.1186/s40635-015-0053-2) contains supplementary material, which is available to authorized users.

## Background

Treatments with bone marrow-derived mesenchymal stem cells (BMSCs) can ameliorate the severity of acute lung injury [1]. Several paracrine factors have also been reported to reduce lung injury. For example, keratinocyte growth factor increases alveolar epithelial fluid clearance [2] and angiopoietin-1 reduces lung vascular permeability [3]. BMSCs can release these paracrine factors, as well as antimicrobial factors [4, 5]. Furthermore, recent studies have shown that direct contact may also be an important condition for the therapeutic mechanisms of BMSCs on acutely injured lung tissue [6]. In that work, mitochondria were transferred from BMSCs to alveolar epithelial cells (AECs) via gap junctions, increasing the functional capacity of AECs and enhancing repair of injured lung tissue.

In the present study, we assessed the production of surfactant and morphological changes in AECs induced by BMSCs co-cultured under in vitro conditions. We examined whether interactions between BMSCs and AECs were responsible for type II AEC phenotype maintenance and induction of AEC morphological rearrangement into three-dimensional alveolar-like structures. We designed both a mixed co-culture model and a separated co-culture model using rat cells to elucidate the effects of direct contact and paracrine factors. We also used these models to assess the effects of BMSCs on surfactant production by type II AECs and on induction of three-dimensional alveolar-like structures.

## Methods

All protocols involving animals were approved by the institutional animal care committee of Tokyo Medical and Dental University.

### Rat bone marrow-derived mesenchymal stem cells

We obtained naïve BMSCs from adult male Sprague–Dawley rats (weight: 180–200 g) using a procedure modified from a previously described study [7]. After deep anesthesia and euthanasia of the rats, the femurs and tibias were extracted bilaterally and the distal epiphyses were removed. An 18-gauge needle was inserted into the proximal end of each bone, and the marrow was flushed into sterile cryovials and stored on ice. The whole bone marrow suspension was cultured on a 10-cm culture dish. The cells were then cultured in Dulbecco’s modified Eagle’s medium (DMEM; Invitrogen, Carlsbad, CA, USA) containing 10 % fetal bovine serum (FBS; Invitrogen) and 1 % penicillin/streptomycin. After 3 days, the tightly adhered cells were trypsinized and resuspended in fresh medium in new culture dishes. The cells were grown to confluence and used at passages 3–10. For cell surface antigen characterization, the BMSC antigens were detected by flow cytometry using anti-rat CD29, CD45, CD54, and CD90 (BioLegend Japan, Tokyo, Japan). The multilineage differentiation potential was assessed by testing the ability of the BMSCs to differentiate into adipocytes, osteoblasts, and chondroblasts, using StemPro Human Mesenchymal Stem Cell Differentiation Kits (Invitrogen). Oil red O, alcian blue, and alizarin red staining was performed to identify adipocytes, chondrocytes, and osteoblasts, respectively. The reproducibility of the experiments was determined using commercially available BMSCs (StemPro Rat Alkali Phosphatase-expressing Mesenchymal Stem Cell, Invitrogen).

### Primary culture of rat alveolar epithelial cells

Primary cultures of rat AECs were prepared as previously described [8]. Briefly, Sprague–Dawley specific pathogen-free 6-week-old rats were tracheostomized under deep anesthesia (125 mg/kg of pentobarbital, intraperitoneal injection), then euthanized by exsanguination. The lungs were removed *en bloc*. After bronchoalveolar lavage with Hank’s balanced salt solution (Invitrogen), the lungs were treated with elastase (Elastase, Porcine Pancreas, Lyophilized, Worthington Biochemical, Lakewood, NJ, USA). The lung tissue was then minced and filtered through 140- and 30-μm nylon mesh filters. The filtered cells were centrifuged, and the cell pellet was resuspended in DMEM and incubated on bacteriological plates at 37 °C in a humidified 5 % CO_2_ water-jacketed incubator for 90 min to reduce contamination with fibroblasts, macrophages, and neutrophils. Unattached cells were collected, and cell purity was assessed by Papanicolaou’s stain. Preparations containing >90 % purity were used, and the cells were seeded at a density of 1.8 × 10^6^ cells/cm^2^ (2 × 10^6^ cells/culture insert) on 12-mm Transwell membranes with 0.4-μm pores and a surface area of 1.12 cm^2^ (#3401, Corning International, Tokyo, Japan). The medium was changed every 2 or 3 days and replaced with DMEM containing 10 % FBS, 100 U/mL penicillin, 100 μg/mL streptomycin, and 10 μg/mL gentamicin, unless the cells were treated for an experiment.

### Co-culture of alveolar epithelial cells with bone marrow-derived mesenchymal stem cells or rat lung fibroblast

#### Separated co-culture

On the day before the AECs were prepared, rat BMSCs were plated at a density of 5 × 10^3^ cells/cm^2^ onto the base of the well. After plating the AECs on the culture insert at a density of 1.8 × 10^6^ cells/cm^2^, the medium was changed every 2 or 3 days with fresh DMEM containing 10 % FBS for 7, 14, or 21 days, while avoiding contamination between the media in the upper and lower wells. As control experiments, adult rat lung fibroblasts (RLFs; Cell Applications Inc., San Diego, CA, USA) were seeded in place of rat BMSC at the same density.

#### Mixed co-culture (AECs on BMSCs)

On the day before the AECs were prepared, rat BMSCs were plated at a density of 2 × 10^5^ cells/cm^2^ on the 12-mm Transwell culture insert. For culture longer than 15 days, the BMSCs were seeded on a collagen type I gel (Rat Tail Collagen I, Invitrogen) at 4 mg/mL to enhance cell attachment. After plating the AECs at a density of 1.8 × 10^6^ cells/cm^2^ (2 × 10^6^ cells/Transwell), the medium was changed every 2 or 3 days with fresh DMEM containing 10 % FBS for 7 or 21 days, while avoiding contamination between the media in the upper and lower wells. As a control, AECs were cultured on a collagen type I gel without first seeding BMSCs for 21 days using the same media or with initial seeding adult RLFs at the same density as rat BMSCs.

### mRNA extraction and real-time polymerase chain reaction

To determine the expression of specific markers for AECs, real-time polymerase chain reaction (PCR) was performed using LightCycler 480 System (Roche Diagnostics Japan, Tokyo, Japan). The total RNA was isolated from AECs cultured on Transwells for 7 and 21 days using a silica membrane column (High Pure RNA Isolation Kit, Roche Diagnostics, Mannheim, Germany). cDNA was synthesized from the total RNA using a Transcriptor First Strand cDNA Synthesis Kit (Roche Diagnostics Japan). The expression of surfactant protein (SP)-A, SP-B, SP-C, and SP-D; receptor for advanced glycation end-products (RAGE); keratinocyte growth factor (KGF); and beta-actin was analyzed by real-time PCR using a LC480 Probe Master mix (Roche Diagnostics Japan). For siRNA experiments, cells were lysed with CellAmp® Direct RNA Prep Kit for reverse-transcription (RT)-PCR (Takara Bio Inc., Shiga, Japan), and the cell lysates were analyzed using a One Step SYBR® PrimeScript® PLUS RT-PCR Kit (Takara Bio Inc.). The primers were designed as shown in Table 1. Data analysis was performed by LightCycler 480 Software (version LCS480 1.5.0.39, Roche Diagnostics Japan), and the amount of expression was normalized to the amount of beta-actin mRNA expression.

**Table 1:** Primer sequences for the real-time PCR analyses

Target	Sequence	
Surfactant protein A (Sftp-A)		
	Forward	CCTGGAGAACGTGGAGACA
	Reverse	GTTTGATCTCATAGAGTTCAGTCTGG
Surfactant protein B (Sftp-B)		
	Forward	TCTGCAATGCTTCCAAACC
	Reverse	GGTCCTTTGGTACAGGTTGC
Surfactant protein C (Sftp-C)		
	Forward	CAAAATGGACATGGGTAGCA
	Reverse	AGAAGGCGTTTGAGATGCAC
Surfactant protein D (Sftp-D)		
	Forward	CACGGAGGGCAAGTTCAC
	Reverse	CCCTGGAGCCCAGTTAGAAT
Receptor for advanced glycation end-products (Ager)		
	Forward	TGTCAACATCAGGGTCACAGA
	Reverse	TCCCTAAGGCCAGGGCTA
β-actin (Actb)		
	Forward	CCCGCGAGTACAACCTTCT
	Reverse	CGTCATCCATGGCGAACT
Keratinocyte growth factor		
	Takara RA050132 (Takara Bio Inc., Shiga, Japan)	

### Immunocytochemistry

After being co-cultured with BMSCs for 7–21 days, the rat AECs on the Transwell were fixed with 4 % formaldehyde in phosphate-buffered saline (PBS), permeabilized with 0.2 % Triton X (Sigma Aldrich Japan, Tokyo, Japan) for 15 min, and incubated in blocking solution containing 1 % bovine serum albumin (Kirkegaard & Perry Laboratories, Gaithersburg, MD, USA) for 30 min. After immunostaining, the Transwell membranes were mounted on slides and imaged by confocal laser scanning microscopy (LSM510 Carl Zeiss MicroImaging, Heidelberg, Germany). The images were processed using Zeiss LSM Image Browser 4.2. (Carl Zeiss MicroImaging) and Zeiss Zen 2011 version 7.0 (Carl Zeiss MicroImaging). For immunostaining, anti-SP-D monoclonal antibody (Acris Antibodies GmbH, Hiddenhausen, Germany), anti-mouse RAGE polyclonal antibody (AF1179, R&D Systems, Minneapolis, MN, USA), and anti-rabbit zona occludens protein 1 antibody (ZO-1, Invitrogen) were used. Alexa Fluor 568 donkey anti-goat IgG, Alexa Fluro 647 chicken anti-rabbit IgG, and Alexa Fluor 488 chicken anti-mouse IgG (Molecular Probes, Carlsbad, CA, USA) were used as secondary antibodies. Four independent experiments were performed for each experimental condition.

### Transmission electron microscopy

After the rat AECs were co-cultured on BMSCs for 14 days on the Transwells (#3413, Corning International), the cells on the membrane of the culture insert were fixed with 2 % glutaraldehyde, 0.8 % paraformaldehyde, and 0.1 M cacodylate. The cells were stained with osmium and imidazole, dehydrated in graded concentrations of ethanol, and then embedded in Epon epoxy resin. Ultrathin sections of the specimens were cut and examined using a transmission electron microscope (TEM H-7100, Hitachi High-Tech, Tokyo, Japan).

### Phospholipid analysis

To assess the effects of the BMSCs on surfactant synthesis, phospholipid production by the primary rat AECs co-cultured with BMSCs was measured using a LipidTOX phospholipidosis detection reagent (Invitrogen). For the rat AECs and BMSCs co-cultured in the separated co-culture technique described above, LipidTOX was added to the upper well of the Transwell, where the AECs were plated, on the day before imaging. After overnight incubation, images were obtained on a fluorescence microscope (Leica DMI4000B, Leica Microsystems, Wetzlar, Germany) after 7, 14, and 21 days of culture.

### KGF knockdown using siRNA

Three days before starting the co-cultures, BMSCs were plated at a density of 1 × 10^4^ cells/cm^2^ in each well of a 12-well Transwell culture insert. After incubation in DMEM with 10 % FBS at 37 °C with 5 % CO_2_ overnight, the cells were cultured in Accell Delivery Media containing 200 nM of siRNA (Accell Rat Fgf7 (29348) siRNA—SMARTpool #K-005000-G1-03 Thermo Scientific Japan, Kanagawa, Japan) for 72 h. Next, the freshly isolated AECs were seeded over these BMSCs, and the co-cultures were maintained in DMEM with 10 % FBS for 48 h. After another treatment with siRNA for 72 h, the cells were harvested for subsequent quantification of transcripts and immunocytochemistry.

### Role of connexin-43 in the alveolar-like structure formation using Gap26 connexin-43-inhibiting peptide

The day before starting the co-cultures, cells were plated at a density of 1 × 10^4^ cells/cm^2^ in each well of a 12-well Transwell culture insert. After incubation in complete media supplemented with 300 μM Gap26 connexin-43-inhibiting peptide (AnaSpec, Fremont, CA, USA) at 37 °C with 5 % CO_2_ overnight, freshly isolated AECs were seeded onto the BMSCs, and the co-cultures were maintained in complete media with Gap26 (300 μM) for 48 h. After another treatment with Gap26 for 72 h, the cells on the culture insert were fixed with 4 % formaldehyde in PBS for subsequent immunocytochemistry.

### Statistical analyses

All statistical analyses were performed using STATA/IC software version 11 (StataCorp, College Station, TX, USA). For comparisons between two groups, Mann–Whitney *U*-tests were used. *P* values less than 0.05 were considered statistically significant.

## Results

### Characterization of rat bone marrow-derived stem cells

The flow cytometry results demonstrated that the rat BMSCs were negative for expression of CD45 and CD54 and positive for CD29 and CD90 (Additional file 1: Figure S1A). In the differentiation experiment, cells were positive for adipogenesis (Additional file 1: Figure S1B), chondrogenesis (Additional file 1: Figure S1C), and osteogenesis (Additional file 1: Figure S1D) after culture with the appropriate induction media. The same characteristics were observed in the StemPro rat BMSCs (data not shown).

### Effect of BMSCs on the cultured alveolar epithelial cells in the separated co-culture

**Fig. 1:**
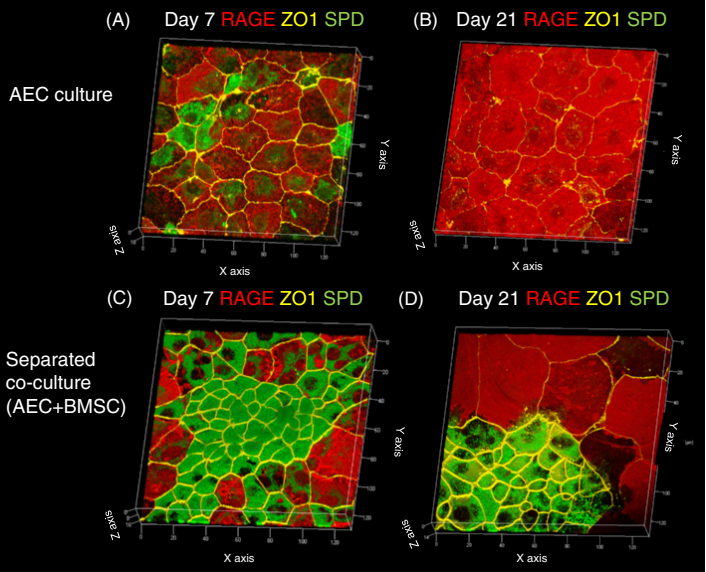
Three-dimensional reconstruction of images of cultured primary AECs on a Transwell insert with (**c, d**) or without (**a, b**) co-culture of BMSCs in the bottom well (separated co-culture) on days 7 (**a, c**) and 21 (**b, d**). *Red staining* indicates expression of RAGE, *yellow staining* indicates ZO-1 expression, and *green staining* indicates expression of SP-D

We cultured primary AECs on the Transwell for 21 days. Representative images of the cultures are shown at day 7 (Fig. 1a) and day 21 (Fig. 1b). In the AEC culture condition without BMSCs, epithelial junctions positive for ZO-1 were established by day 7, and both SP-D-positive and RAGE-positive cells were observed on that day. However, SP-D expression had decreased by day 21 (Fig. 1b).

**Fig. 2:**
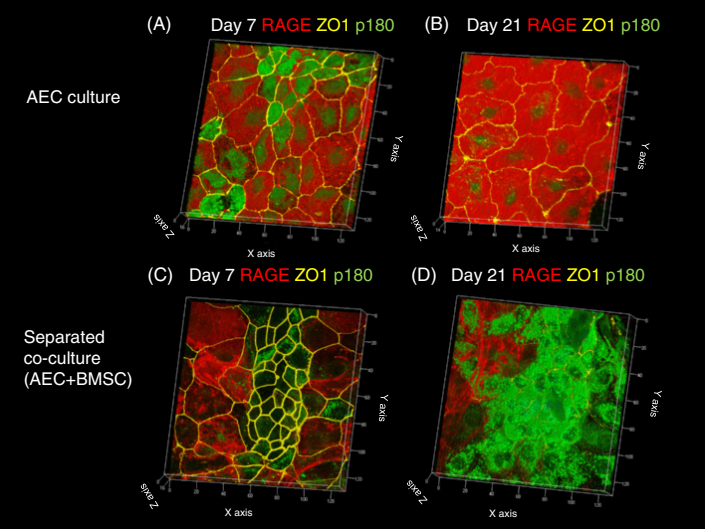
Three-dimensional reconstruction of images of cultured primary AECs on a Transwell insert with (**c, d**) or without (**a, b**) co-culture of BMSCs in the bottom well (separated co-culture) on days 7 (**a, c**) and 21 (**b, d**). *Red staining* indicates expression of RAGE, *yellow staining* indicates ZO-1 expression, and *green staining* indicates expression of p180

**Fig. 3:**
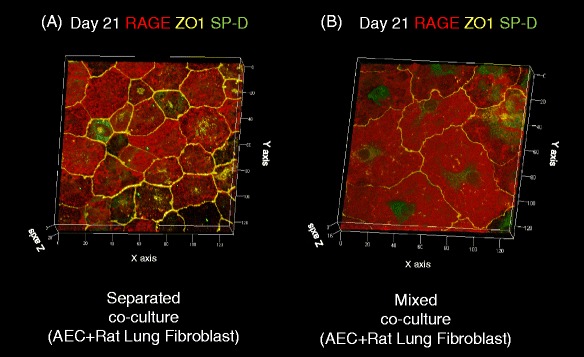
Three-dimensional reconstruction of images of primary AECs co-cultured with rat lung fibroblasts. **a** Separated co-culture on day 21 and **b** mixed co-culture on day 21. *Red staining* indicates expression of RAGE, *yellow staining* indicates ZO-1 expression, and *green staining* indicates expression of SP-D

**Fig. 4:**
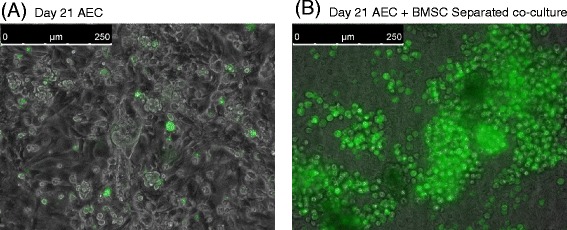
Phospholipid staining of primary AECs cultured on a Transwell on day 21. **a** Primary culture of AECs alone with isolated phospholipid-positive cells indicated in *green*. **b** Separated co-culture of AECs with BMSCs induced cluster formation of phospholipid-positive cells indicated in *green*

However, the separated co-culture condition showed cluster formation of SP-D-positive cells from day 7 to day 21 (Fig. 1c, d). After changing from the anti-SP-D antibody to the anti-p180 lamellar body protein, the cells remained p180-positive in the separated co-culture, but p180 expression was decreased by day 21 in the ATII cells cultured without BMSC co-culture (Fig. 2a–d). Separate co-culture of AEC with rat lung fibroblasts did not induce cluster formation of SP-D-positive cells on day 21 (Fig. 3a). We then tested whether these type II-like cells demonstrated surfactant production by staining the cultured primary cells with LipidTOX phospholipid detection reagent. Although the control AEC culture without BMSCs showed a very small number of phospholipid-positive cells on day 21 (Fig. 4a), the co-culture with BMSCs demonstrated abundant cluster formation of phospholipid-positive cells (Fig. 4b) at the same time point.

**Fig. 5:**
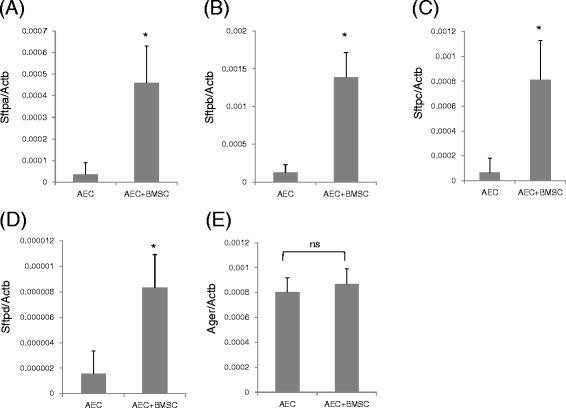
mRNA expression levels of surfactant proteins in AECs. Expression of **a** SP-A (Sftpa), **b** SP-B (Sftpb), **c** SP-C (Sftpc), **d** SP-D (Sftpd), and **e** RAGE (Ager) in cultured primary AECs with or without co-culture of BMSC on day 21. <<AEC>>: AECs without co-culture with BMSC; <<AEC + BMSC>>: AECs co-cultured with BMSCs. Levels of each transcript were determined by real-time PCR and normalized to those of beta-actin (Actb). *P < 0.05 vs. AEC; *ns* not significant

The transcripts of SP-A (Sftpa), SP-B (Sftpb), SP-C (Sftpc), and SP-D (Sftpd) were significantly increased (*P* < 0.05) in the separated co-culture condition using BMSCs on day 21 compared with the culture condition without BMSCs (Fig. 5a–d), whereas the transcript of RAGE (Ager) showed no change (Fig. 5e).

### Effect of BMSCs on the cultured alveolar epithelial cells in the mixed co-culture

**Fig. 6:**
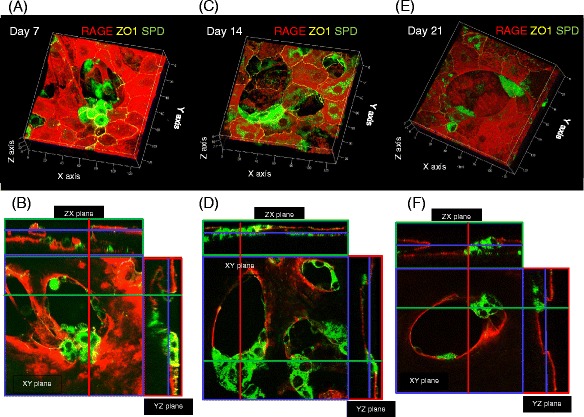
Three-dimensional images of AECs in direct contact with BMSCs. **a, c, e** Three-dimensional reconstructions of images of primary AECs co-cultured in direct contact with BMSCs. Cells were stained with antibodies against RAGE (*red*), SP-D (*green*), and ZO-1 (yellow). **b, d, f** Orthogonal section analysis of the images in **a**, **c**, and **e**. Images of AECs on Transwell culture inserts on days 7 (**a, b**), 14 (**c, d**), and 21 (**e, f**). *Green*, *red*, and *blue rectangles* indicate sections of the images at the level of the line with the corresponding color

Without co-culture of BMSCs, RAGE-positive AECs covered the surface of the collagen type I gel on day 21. In this culture condition, most of the AECs expressed RAGE, but not SP-D. In contrast, in the mixed co-culture condition, BMSCs induced the formation of alveolar-like structures on days 7, 14, and 21 (Fig. 6a–f, Additional file 2: Movie). The surface of these structures was covered with RAGE-positive AECs, and some of the cells were also positive for SP-D. In some preparations, clusters of the SP-D-positive cells were observed (Fig. 6a–f, Additional file 2: Movie), and these SP-D-positive cells were shaped like cuboidal cells with ZO-1 expression at the apical side of the cellular junctions (Fig. 6b, d, f, Additional file 2: Movie). However, mixed co-culture of AEC with rat lung fibroblast yielded no three-dimensional alveolar-like structure formation on day 21 (Fig. 3b).

**Fig. 7:**
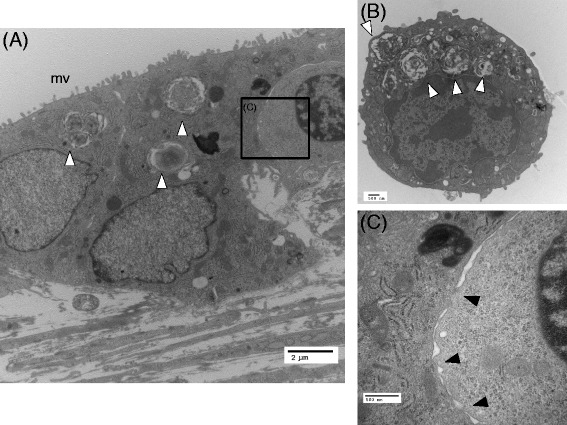
Transmission electron micrographic images of AECs in direct contact with BMSCs on day 14. **a** Type II-like epithelial cells observed in this preparation. **b** Freshly isolated type II AECs. The lamellar bodies that were observed in the freshly isolated cells were also observed in some of the epithelial cells in **a** (**a, b** *white arrow heads*). **c** A magnified image of the cellular contact observed in the *black rectangle* shown in **a**. The *black arrow heads* indicate contacts between the epithelial and mesenchymal cells. *mv* microvilli

**Fig. 8:**
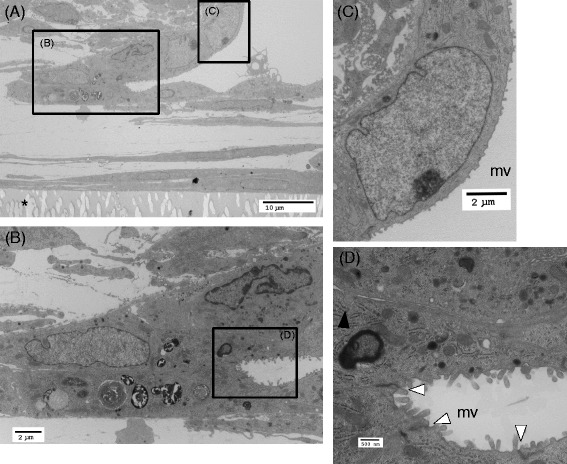
Transmission electron micrographic images of the alveolar-like structures observed in cultures of AECs in direct contact with BMSCs on day 14. **a** Alveolar-like structures consist of mesenchymal cells and matrix on the culture insert (asterisk) covered with an epithelial cell monolayer. **b** A magnification of a rectangle shown in **a**. At the edge of the bottom of the alveolar-like structures, type II-like cells with lamellar bodies are shown. **c** Cells with type I-like phenotype. Magnification of the rectangle shown in **a**. **d** A magnification of the rectangle shown in **b**. *White arrow heads* indicate tight junctions between epithelial cells, and the *black arrow head* indicates a cellular contact between the mesenchymal and epithelial cells via a small process

Transmission electron micrographs of the mixed co-cultures on day 14 showed that the mesenchymal cells were covered by an epithelial monolayer, and some of the epithelial cells had lamellar body formation (Fig. 7a), which was similar to that of freshly isolated type II AECs (Fig. 7b). The same structures as those shown in Fig. 6 were observed in the transmission electron micrographs (Fig. 8a–d), and cells with lamellar bodies (Fig. 8b) were observed with type I-like epithelial cells (Fig. 8c), which covered the collagen-rich mesenchymal cells. Some epithelial cells were in contact with the mesenchymal cells via small processes (Figs. 7c and 8d).

**Fig. 9:**
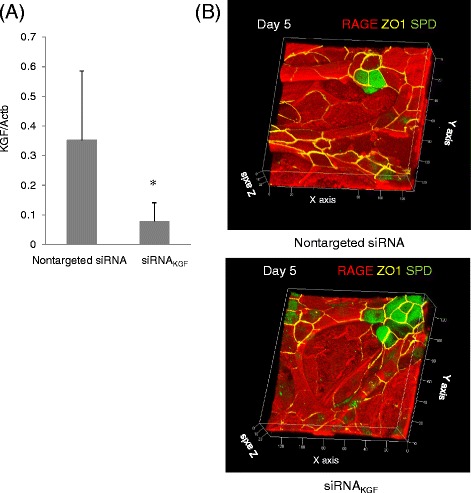
Effect of siRNA knockdown of KGF on AECs cultured on BMSCs. **a** Comparison of the abundance of KGF transcript between cells treated with siRNA for KGF expression (siRNA_KGF_) and cells treated with non-target siRNA (Control). *P < 0.05 vs. non-target siRNA. **b** Three-dimensional images of AECs in direct contact with BMSCs with or without KGF knockdown. *Upper panel* shows cells treated with non-target siRNA, and the *lower panel* shows cells treated with siRNA_KGF_. Cells were stained with antibodies against RAGE (*red*), SP-D (*green*), and ZO-1 (*yellow*)

**Fig. 10:**
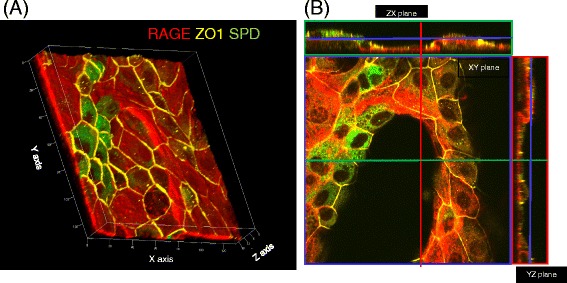
Effect of Gap26 on AECs cultured on BMSCs. **a** Three-dimensional images of AECs in direct contact with BMSCs treated with Gap26. **b** Orthogonal section analysis of image. *Green*, *red*, and *blue rectangles* show the sectional images at the level of the line with the corresponding color. Cells were stained with antibodies against RAGE (*red*), SP-D (*green*), and ZO-1 (*yellow*)

To determine the effect of KGF on the formation of this type of structure, we treated the mixed co-cultures with siRNA for KGF. In this preparation, the transcript for KGF expression was knocked down to 22 % of that by cells treated with non-targeted siRNA (Fig. 9a). While siRNA treatment caused a significant inhibition of surfactant protein transcript expression (reduction of 77 ± 20 %, 88 ± 11 %, 69 ± 13 %, and 39 ± 15 % in SP-A, SP-B, SP-C, and SP-D, respectively), formation of the alveolar-like structure was not affected (Fig. 9b). We also assessed whether the functional inhibitor of connexin-43, Gap26, could mitigate the effect of BMSCs on AECs. However, formation of the alveolar-like structure was not affected following the use of up to 300 μM Gap26 (Fig. 10a, b).

## Discussion

Alveolar epithelial phenotypes were maintained for 3 weeks of culture under separated co-culture conditions with BMSCs. Additionally, this co-culture induced cluster formation of SP-D-positive, type II AEC-like cells. Phospholipid staining also revealed formation of phospholipid-positive cell clusters, suggesting that paracrine factors augment production of surfactant-related substances in AECs. In mixed co-culture conditions, BMSCs induced formation of alveolar-like structures, and AECs were in direct contact with BMSCs via small processes. This indicates a direct interaction between these cells. Formation of the alveolar-like structure was not affected by either KGF knockdown or connexin-43 inhibition. These results suggest that the type II AEC phenotype can be maintained via paracrine factors released by BMSCs, and direct contact between AECs and BMSCs induced morphological changes which lead to the formation of alveolar-like structures. Although the duration of BMSC phenotype maintenance in these co-culture models is unknown, interactions between AECs and BMSCs could facilitate maintenance of type II AEC phenotypes and promote specific three-dimensional structure formation.

In most culture conditions on tissue culture plastic, type II AECs tend to differentiate into type I AECs. Consequently, it has proven difficult to maintain surfactant-producing cells [9]. The AEC cultures without BMSC co-culture showed flat monolayers mostly consisting of cells with type I phenotype, and this result was consistent with previous reports. However, separated co-culture resulted in 1) cluster formation of SP-D- and p180 lamellar body protein-positive cells, 2) cluster formation of phospholipid-positive cells, and 3) increased expression of transcripts for SP-A, B, C, and D until day 21 of culture.

These results could be attributed to either proliferation of type II epithelial cells or delayed trans-differentiation of type II to type I AECs. Furthermore, the increased production of surfactant proteins by type II phenotype cells suggests that BMSCs released paracrine factors that contributed to the maintenance and/or proliferation of cells with a type II alveolar epithelial phenotype. KGF is an important paracrine factor required for induction of pulmonary surfactant protein synthesis in both fetal and adult lungs [10–12] and therefore could be one of the paracrine factors released by BMSCs.

In the mixed co-culture condition, the BMSCs induced alveolar-like structure formation that was maintained until day 21 of culture. This alveolar-like structure had several characteristics resembling those observed in the alveolar-like structures in the normal lung tissue. First, the alveolar-like structures were covered with a monolayer of type I and type II AECs. Small clusters of type II AECs were frequently observed at the invagination of the epithelial layer, which resembled the location of the type II AECs frequently found in the depressions of the alveolar wall. Second, some of the cells had small processes that contributed to the intercellular communication between the BMSCs and AECs. This feature was similar to the intercellular contacts between type II AECs and the fibroblast seen in the normal human lung tissue [13]. We examined whether KGF and connexin-43 contributed to alveolar-like structure formation, but these factors did not appear to be involved. Previous studies identified expression of connexin-43 and modified Ca^2+^ communication in rat alveolar type I and type II epithelial cells [14]. Connexin-43 is expressed in human BMSCs [15] and mouse BMSCs [6]. Moreover, Islam et al. have shown that mitochondria containing microvesicles were transferred between BMSCs and AECs via connexin-43-containing gap junctions using a mouse acute lung injury model [6]. However, our present results suggest that cellular interaction via connexin-43 was not required for formation of the three-dimensional alveolar-like structure. A recent study found that a certain basement membrane matrix protein specialized for three-dimensional culture can induce the formation of alveolar cyst-like structures by AECs [16]. Given that our mixed co-culture model was maintained using only DMEM and collagen, we suspect that the BMSCs or the matrix molecules released by the BMSCs may act as substitutes for these types of matrices in three-dimensional culture.

Currently, the engraftment rate of BMSCs in lung injury models is reported to be low [17–19]. Therefore, the contribution of a mechanism related to the direct contact between BMSCs and AECs might be less important in vivo than in the present in vitro culture model. However, alveolar-like structure formation in our mixed co-culture model occurred during the early phase of culture, and there were no further structural developments after day 7. This result could be explained if both AECs and BMSCs have their own interactive regulations that become stabilized once the cells attach to the tissue matrix. Supposing that BMSCs reach injured and denuded interstitial tissue, there remains a possibility that direct contact between BMSCs and AECs facilitates the stabilization of the AECs in vivo*.* However, these hypotheses should be verified in the experiments using injured epithelial cells or in in vivo lung injury models.

There are several limitations to this study. First, because of the difficulty of isolating pure AECs, there may have been a small fraction of fibroblastic cells included in the AEC populations. These fibroblasts could then be stimulated by paracrine factors released by the BMSCs. However, neither the AEC- alone nor AEC-lung fibroblast co-culture conditions demonstrated alveolar-like structure formation. Therefore, we believe that direct contact between the BMSCs and AECs was required for induction of three-dimensional structure formation. Second, we did not assess the phenotype of the co-cultured BMSCs on day 21 of culture. Subsequently, we cannot confirm that BMSCs maintained their phenotype through until completion of experiments. Therefore, it is possible that these cells underwent phenotype changes as a result of culture conditions or interactions with AECs. Third, considering that intratracheally administered BMSCs reach the alveoli where they contact injured AECs covering alveolar surface, mixed co-culture model should be designed as the one in which BMSCs are seeded on the AEC monolayer on type I collagen (BMSCs on AECs). In the current study, we supposed that the injured alveoli in which AECs were denuded have interstitial tissue exposed to alveolar space. Our hypothesis is that BMSCs support functions of alveolar epithelial type II cells and induce reforming the appropriate alveolar structure, which result in the recovery of function of alveolar tissue in the lung. Furthermore, considering the route of administration of BMSC, it is not limited to the airway, and intravenous administration might be a practical option. In this context, BMSCs reach to the interstitial tissue and cause some effect. The current study design is based on these theoretical backgrounds, and further study is needed to unveil the interaction between BMSCs and AECs when BMSCs reached on the AECs covering alveolar space in the lung. Fourth, there is also a possibility that that the degree of KGF knockdown, or concentrations of Gap26 employed were not sufficient to inhibit formation of three-dimensional alveolar-like structures. Additionally, other gap junction-related proteins that are not inhibited by Gap26 may have been involved in three-dimensional structure formation. Fifth, our results are based on in vitro culture systems. While common features exist between in vivo tissue structures and the alveolar-like structures generated, there are also differences. For example, the current in vitro culture model did not contain endothelial cells. Moreover, recent three-dimensional culture systems could facilitate formation of complex three-dimensional structures. Finally, the present findings were obtained using rat-derived cells, and interspecies differences should be considered. Future investigations using human cells are required to confirm whether similar effects can be induced by BMSCs in human models.

## Conclusions

BMSCs release soluble paracrine factors that maintain the surfactant releasing potential and a type II-like AEC phenotype for a sustained period in the separated co-culture model. Furthermore, a direct interaction between BMSCs and AECs may induce formation of cyst-like three-dimensional structures covered with AECs. These characteristics of BMSCs could aid therapeutic strategies to restore alveolar structure and function in the lung tissue following injury through interaction with residual AECs.

## Additional files

Additional file 1: Figure S1. Characterization of BMSCs prepared from rat tibias and femurs. (A) Surface expression levels of CD29, CD45, CD54, and CD90 were analyzed by flow cytometry. In each panel, the red line represents the counts of cells stained with an antibody for each indicated antigen, while the blue line shows the number of cells treated with an isotype control IgG. (B) Induction of adipogenic differentiation. Cells positive for adipogenesis are stained with oil red O. (C) Induction of chondrogenic differentiation. Cell pellets positive for chondrogenesis are stained with alcian blue. (D) Cells positive for osteogenesis are stained with alizarin red S. Additional file 2: Movie
**Three-dimensional reconstruction images of primary AECs co-cultured in direct contact with BMSCs.** Image depicts AECs on the culture insert of a Transwell on day 7, rotating about the *y*-axis to demonstrate the three-dimensional alveolar-like structure. Cells were stained with antibodies against RAGE (red), SP-D (green), and ZO-1 (yellow).

## Abbreviations

BMSCs: bone marrow-derived mesenchymal stem cells
AECs: alveolar epithelial cells
DMEM: Dulbecco’s modified Eagle’s medium
FBS: fetal bovine serum
SP-A, -B, -C, -D: surfactant protein A, B, C, D
RAGE: receptor for advanced glycation end-products
ZO-1: zona occludens protein 1
KGF: keratinocyte growth factor
