# Supplementary figures and images for: Interactions between rat alveolar epithelial cells and bone marrow-derived mesenchymal stem cells: an in vitro co-culture model
Source: Intensive Care Med Exp. 2015 May 24;3:15. doi: 10.1186/s40635-015-0053-2 (PMC4480799; doi:10.1186/s40635-015-0053-2)

(A)

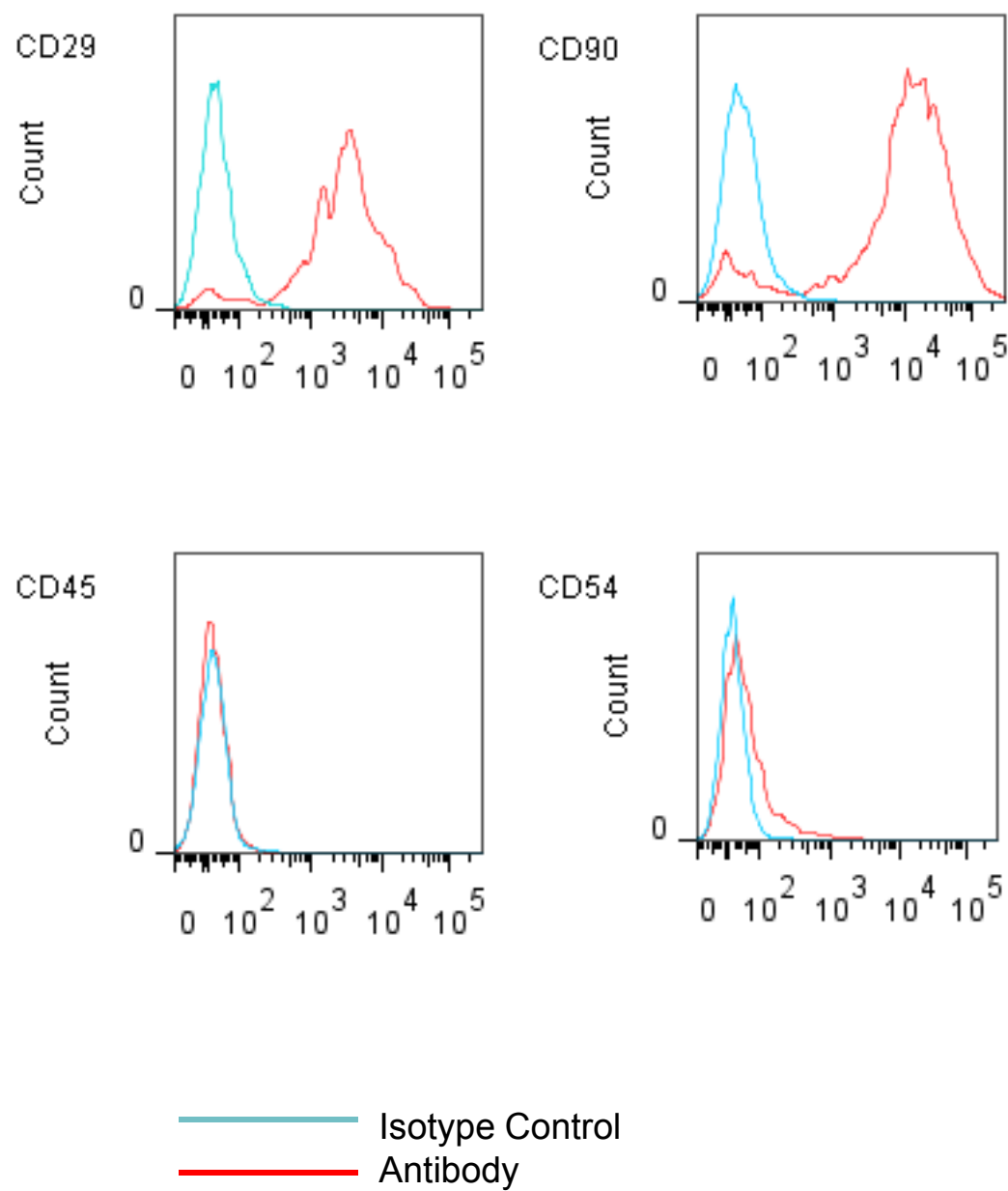

(B)

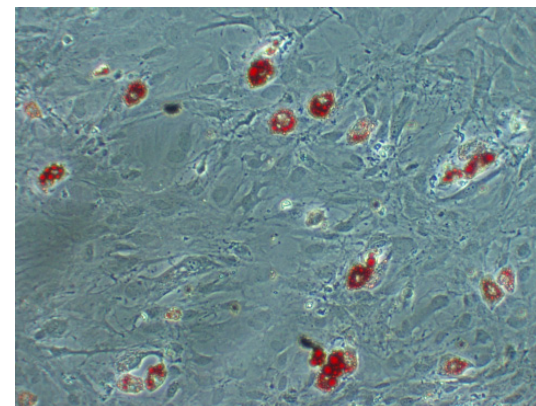

Adipogenesis

(C)

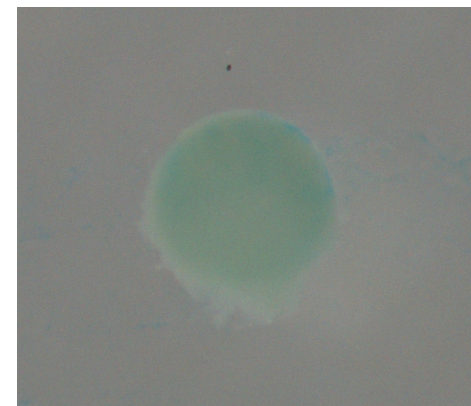

Chondrogenesis

(D)

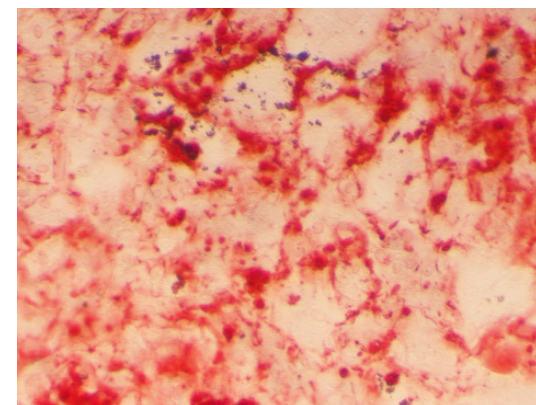

Osteogenesis

Supplement: Additional file 1: Figure S1. — Characterization of BMSCs prepared from rat tibias and femurs. (A) Surface expression levels of CD29, CD45, CD54, and CD90 were analyzed by flow cytometry. In each panel, the red line represents the counts of cells stained with an antibody for each indicated antigen, while the blue line shows the number of cells treated with an isotype control IgG. (B) Induction of adipogenic differentiation. Cells positive for adipogenesis are stained with oil red O. (C) Induction of chondrogenic differentiation. Cell pellets positive for chondrogenesis are stained with alcian blue. (D) Cells positive for osteogenesis are stained with alizarin red S. [file 40635_2015_53_MOESM1_ESM.pdf]
